# Establishing childhood disability clinics may help reduce the prevalence of disability among children in Africa: A viewpoint

**DOI:** 10.3389/fpubh.2022.1010437

**Published:** 2022-11-04

**Authors:** Auwal Abdullahi, Thomson W. L. Wong, Shamay S. M. Ng

**Affiliations:** Department of Rehabilitation Sciences, The Hong Kong Polytechnic University, Kowloon, Hong Kong SAR, China

**Keywords:** children, disability, Africa, quality of life, sustainable development goal

## Abstract

Globally, there are about a billion people comprising of about 95 million children who experience disability. The number of people in Africa living with disability is about 80 million people; out of which 10%−15% are children of school age. The causes of disability among these children include epilepsy, vision loss, or hearing loss, cerebral palsy, poliomyelitis, tetanus, cerebrospinal meningitis and malaria. However, these causes of disability are preventable and can be managed with proper care. The aim of this article is to propose the establishment of childhood disability clinics in Africa in order to help prevent or reduce the incidence/ prevalence of disability among children. Some of the mandates of the clinics will be to carry out routine assessment of children for disability, to provide education on disability and strategies for disability prevention to parents and caregivers, to promptly prevent and manage disability or its causes. However, establishing these clinics requires shared commitment of all the stakeholders.

## Introduction

According to the UN Convention on the Rights of Persons with Disabilities (UNCRPD), disability refers to having impairments in physical, mental, intellectual or sensory functions that can hinder one's full and effective participation in the society on equal basis with others (1). Statistics indicates that, about 14%−15%, which equals to about a billion people of the world's population, experience one form of disability or the other (2, 3). This statistics could be much higher when the families of those living with disabilities are considered since they also bear the burden of their loved ones with disabilities (4).

The prevalence is however higher in the developing countries (3). This is attributed to the increased prevalence of chronic health conditions, violence and conflict, and delay in accessing health services (5, 6). Among children, prevalence of disability ranges between 0.4% and 3%, and is higher in boys (7). Specifically, the Global Burden of Disease estimates childhood disability prevalence to be 95 million (5.1%) children, of whom 13 million (0.7%) has severe disability (3). However, the higher prevalence in boys compared to girls, has been argued to be due to lack of universal criteria for defining disability types such as learning disability, and genetic factors such as in the case of autism spectrum disorder (8, 9).

Although, estimating the extent of disability among communities is a herculean task (10); it is estimated that there are about 80 million people living with disability in Africa (11, 12). Out of this number, 10%−15% are children of school age (11, 12). Causes of disability among children and adolescents include epilepsy, cerebral palsy, poliomyelitis, tetanus, cerebrospinal meningitis, malaria and limb amputation (3, 6, 13).

## The viewpoint

### The problem in focus

Childhood is supposed to be a sweet experience. This sweet experience may be shattered by having a disability (14). This is because people living with disabilities have a higher incidence of chronic conditions and health-related disparities (15, 16). Chronic conditions in turn can also cause disability, which can also increase the risk for other conditions (5).

In Africa, the commonest cause of disability is infection, followed by trauma due to accidents or war, congenital and non-infectious diseases such as epilepsy, poor quality of perinatal care, malnutrition, and chronic medical conditions such as diabetes and HIV/AIDS (17–20). Infants who are HIV-positive have increased prevalence of developing neurological problems such as movement and cognitive impairment that can result in disability (17).

When a person has disability, they may experience physical challenges, cognitive problems, stigmatization, loss of employment opportunity, poor productivity and economic loss, and impose caregiver burden and economic burden to families and government (3, 7, 12, 21). For instance, a child with lower limb amputation may find it difficult to walk and carry out his activities of daily living (ADL). Similarly, a child with cognitive problem may find it difficult to pay attention in class to learn; or learn social skills that can help them relate with peers and other people. Thus, it is imperative that causes of disability and disability during childhood are recognized and dealt with promptly.

Unfortunately, often times due to the nature of the health systems in Africa, healthcare professionals see children in the clinics only when they present with serious health problems. However, most health problems in children such as impairment in movement, cognitive ability and social skills development become obvious only when the children are growing up (22, 23). As a result of this, only very skilled healthcare professionals or special educationists may be able to observe that something is wrong with the child. Therefore, even though, there are postnatal and children clinics in Africa, these clinics do not specifically focus on disability assessment and prevention. As such, there is a need for clinics that will specifically focus on routine assessment of children for impairment and/ or disability even if they are apparently healthy at least once every month. The aim of this paper is to provide an argument for establishing childhood disability clinics in Africa.

### The mandates of the clinics

The mandates of the clinics will be to provide many services related to disability prevention and treatment such as the following:

#### Routine assessment of children for disability

Assessments for physical and cognitive health, and social skills development should be carried out. Through these assessments, existing and potential problems can be recognized or predicted. Consequently, training and advice can be offered to the parents/caregivers. In addition, services such as surgical or medical treatments, rehabilitation, special education and nursing can be provided. Assessment for disability can help improve outcome in people living with disabilities (24). This is because knowing a problem is half way to solving it. For instance, if it is realized that a child has weakness of the lower limb, the muscles can be strengthened or they will be provided with an orthotic device such as the calipers. Similarly, if a child has hearing problem, hearing aids or appropriate services can be provided promptly.

However, for assessment of disability among children to be effective, it needs to be comprehensive and carried out at many different intervals, not once (25). That is to say, the assessment should be carried out routinely. Some of the factors that will ensure effective and comprehensive routine assessment of children with disabilities include the organizational structures, resources, therapists within the organizations, assessment tools, and families of members of the children (26). Therefore, utilizing these factors appropriately can help in supporting effective assessment of children with disabilities. Consequently, the WHO International Classification of Functioning, Disability and Health Child and Youth Version Activities and Participation D Codes can be used for the assessment (25). If after the assessment, a child is discovered to have a certain disability, prompt intervention should be given in order to ensure the child enjoy a full and decent life, in conditions which ensure dignity, promote self-reliance and facilitate the child's active participation in the community, according the UN Convention on the Rights of the Child (27).

#### Disability education for parents and/or caregivers

This should involve education on the misconception of causes of disability. In many parts of the world, many people still believe that disability is caused by spiritual attack or the fault of the parents (28). This can affect readiness of families to seek for right help for their children. In addition, provision of training and offering advice to parents and caregivers on how to prevent and/ or manage conditions or symptoms such as fever, recognize adverse events such as fever, swelling and pain following immunization, and prevent falls and its consequential injuries should also be given priority. For this, a manual can be provided after the training to help easily guide the parents/ the caregivers. An example of this is the training manual for caregivers of children living with disabilities developed by the UNICEF (29).

Furthermore, the education and training should include adequate information on disabilities of their children, the help the caregivers can offer on their own and the support they need and where they can get it (21). This can serve as a form of empowerment to their caregivers to become advocates, and can help them get the courage to assist the child with disability to be as independent as they can in carrying out their ADL (21). This is because active engagement of caregivers in the care of their children with disabilities may help improve outcomes (30).

#### Provision of prompt prevention and intervention strategies for childhood disability

This should include treatment and rehabilitation, nursing services, dietary advice, special education and counseling. In addition, play therapy should also be used. Accordingly, there have been calls for action for early intervention services and early childhood development for children living with disabilities (31, 32). These interventions should help minimize the disabling effects of impairments (31). Examples of these can include provision of orthotic devices for children such as those with polio to start using them in order to prevent contracture and further disability. In addition, a child with tetanus infection can be managed using therapeutic positioning to prevent development of joint stiffness and contractures. Similarly, maternal care can be enhanced to help prevent perinatal conditions that can result in disabilities (31). For instance, use of mosquito nets can be encouraged to prevent pregnant women from malarial infection. Malarial infection can affect the developing feotus and cause stillbirth and low birth weight (33, 34). Low birth weight is a predisposing factor for conditions such as cerebral palsy (CP) which can cause disabilities in children (35).

#### Provision of training to those who are providing the services

The African Child Policy Forum (ACPF) recommends capacity building in terms of technical skills and know-how for the stakeholders involved in the care of children with disabilities (31). According to the forum, regular training and support through regular site visits, call centers, resources centers and online support using online materials and phone calls should be provided. This will help to enhance the capacity of the care providers and the clinics as well. In particular, capacity building for healthcare providers who are involved in the caring of people with disability in the low and middle income countries has been emphasized (36). This capacity building should also include training on assessment of disability (37). Building capacity can help improve outcomes in healthcare practice (38).

#### Research and development

Research is the backbone of knowledge. It gives insights about phenomenon or happenings in the world. Thus, through research, the stakeholders will further understand and come up with innovative ways to help prevent or manage disability in children including the needs of the children and their families. However, for any research on children with disabilities, the research should include people living with disabilities in the research team, as it will enhance understanding of the relevance of the findings to all the stakeholders (39).

### Possible composition of the team to manage the clinics

The team shall comprise of the caregivers/parents, clinical psychologists/psychiatrists, dietitians, nurses, pediatricians/surgeons, and rehabilitation professionals such as the physiotherapists, occupational therapists, speech therapists, audiologists and special educationists. Where possible the tasks of routine assessment of the children for disability can be shifted to trained Community Health Officers or Extension Workers or the caregivers. However, to ensure effective task shifting, adequate training needs to be given. When adequate training is given, task shifting is feasible and can result in the success of the task (40).

### Methods of service delivery

The service can be provided in the clinic, the community and through telecare. The advantage of providing the service in the clinic is that, the service can be integrated into the already existing postnatal or pediatric clinic to help conserve resources. Providing the service in the community on the other hand may also have its own advantage since sometimes patients prefer to be treated at home (14, 31). Similarly, telecare can also be used to reach people living with disabilities especially those who are in remote areas. This can help facilitate self-management (41).

### Stakeholders for establishing the clinics

The stakeholders for establishing the clinics should include healthcare professionals, people living with disabilities, parents/caregivers, pressure groups such as the non-governmental organizations, the community and the government. The health professionals should educate the general public including the government on the need to establish such clinics. People living with disabilities, parents/caregivers, non-governmental organizations, and the community can serve as pressure groups that will persuade governments to establish the clinics. In addition, if possible, public/private sector partnership can be used in establishing such clinics when the stakeholders deem that this can give the clinics better prospects. According to Cameroun et al., healthcare professionals who are involved in the care of children with disabilities in low middle income countries (LICs) can get help in many ways (42). For instance, they can receive training and grant supports from many non-governmental organizations such as Médecins Sans Frontières (Doctors without Borders) and the United Nations Children's Fund support programs for children (42).

## Conclusion

Establishing childhood disability clinics in Africa may help prevent or reduce the incidence or prevalence of disability among children. Interestingly, establishing the clinics could be possible since there are already existing postnatal and children clinics in most health facilities. However, this requires shared commitment of all the stakeholders. In addition capacity building of all the stakeholders involved with the clinics is very important. Furthermore, tasks shifting whereby staffs such as the Community Health Officers are trained to carry out some of the mandates of the clinics may help make the service delivery easier.

## Author contributions

All authors contributed to the writing and approved the manuscript for publication.

## Funding

This work was supported by the research funding of the Research Centre for Chinese Medicine Innovation of The Hong Kong Polytechnic University (Ref. No. P0041139) awarded to SN and her team; and PolyU Distinguished Postdoctoral Fellowship Scheme (P0035217).

## Conflict of interest

The authors declare that the research was conducted in the absence of any commercial or financial relationships that could be construed as a potential conflict of interest.

## Publisher's note

All claims expressed in this article are solely those of the authors and do not necessarily represent those of their affiliated organizations, or those of the publisher, the editors and the reviewers. Any product that may be evaluated in this article, or claim that may be made by its manufacturer, is not guaranteed or endorsed by the publisher.
